# Chemical Composition and Assessment of Antimicrobial Activity of Lavender Essential Oil and Some By-Products

**DOI:** 10.3390/plants10091829

**Published:** 2021-09-03

**Authors:** Alexandru Ciocarlan, Lucian Lupascu, Aculina Aricu, Ion Dragalin, Violeta Popescu, Elisabeta-Irina Geana, Roxana Elena Ionete, Nicoleta Vornicu, Octavian G. Duliu, Gergana Hristozova, Inga Zinicovscaia

**Affiliations:** 1 Department of Chemistry of Natural and Biologically Active Compounds, Institute of Chemistry, Academiei Str. 3, MD-2028 Chisinau, Moldova; algciocarlan@yahoo.com (A.C.); lucian1978@mail.ru (L.L.); aculina.aricu@gmail.com (A.A.); iondragalin@yahoo.com (I.D.); violeta.popescu74@gmail.com (V.P.); 2 Department of Research and Development, National Research and Development Institute for Cryogenics and Isotopic Technologies—ICSI Rm. Valcea, 4th Uzinei Str., PO Raureni Box 7, 240050 Rm. Valcea, Romania; irina.geana@icsi.ro (E.-I.G.); roxana.ionete@icsi.ro (R.E.I.); 3 Metropolitan Center of Research T.A.B.O.R., 9 Closca Str., RO-700066 Iasi, Romania; cmctaboriasi@yahoo.com; 4 Department of Structure of Matter, Faculty of Physics, Earth and Atmospheric Physics and Astrophysics, University of Bucharest, 405 Atomistilor Street, 077125 Magurele, Romania; o.duliu@upcmail.ro; 5 Department of Nuclear Physics, Joint Institute for Nuclear Research, 6 Joliot-Curie Str., 141980 Dubna, Russia; gerihris2@gmail.com; 6 Faculty of Physics and Technology, Plovdiv University “Paisii Hilendarski”, 24 Tsar Asen Str., 4000 Plovdiv, Bulgaria; 7 Department of Nuclear Physics, Horia Hulubei National Institute for R&D in Physics and Nuclear Engineering, 30 Reactorului Str. MG-6, 077125 Magurele, Romania

**Keywords:** *Lavandula angustifolia* L., essential oil, by-products, terpenic compounds, chromatographic analyses, antimicrobial activity, statistical data analysis

## Abstract

The producers of essential oils from the Republic of Moldova care about the quality of their products and at the same time, try to capitalize on the waste from processing. The purpose of the present study was to analyze the chemical composition of lavender (*Lavanda angustifolia* L.) essential oil and some by-products derived from its production (residual water, residual herbs), as well as to assess their “in vitro” antimicrobial activity. The gas chromatography-mass spectrometry analysis of essential oils produced by seven industrial manufacturers led to the identification of 41 constituents that meant 96.80–99.79% of the total. The main constituents are monoterpenes (84.08–92.55%), followed by sesquiterpenes (3.30–13.45%), and some aliphatic compounds (1.42–3.90%). The high-performance liquid chromatography analysis allowed the quantification of known triterpenes, ursolic, and oleanolic acids, in freshly dried lavender plants and in the residual by-products after hydrodistillation of the essential oil. The lavender essential oil showed good antibacterial activity against *Bacillus subtilis*, *Pseudomonas fluorescens*, *Xanthomonas campestris*, *Erwinia carotovora* at 300 μg/mL concentration, and *Erwinia amylovora*, *Candida utilis* at 150 μg/mL concentration, respectively. Lavender plant material but also the residual water and ethanolic extracts from the solid waste residue showed high antimicrobial activity against *Aspergillus niger*, *Alternaria alternata*, *Penicillium chrysogenum*, *Bacillus* sp., and *Pseudomonas aeroginosa* strains, at 0.75–6.0 μg/mL, 0.08–0.125 μg/mL, and 0.05–4.0 μg/mL, respectively.

## 1. Introduction

*Lavandula angustifolia* Mill. (syn. *Lavandula vera* DC, syn. *Lavandula officinalis* Chaix ex Vill., syn. *Lavandula spica* L.) is a perennial evergreen shrub of the family *Lamiaceae*, native to the Mediterranean region. Nowadays, this species is naturalized almost all over Europe, North Africa, United States, and Australia [1]. *L.angustifolia* (Lavander) is one of the most valuable medicinal and aromatic plants traditionally used to treat pain, parasitic infections, burns, insect bites, cramps, and muscle spasms [2]. In addition to its application in herbal treatment, lavender is also cultivated for the essential oils used in aromatherapy and the cosmetic, food, and flavour industries [3,4,5].

This is possible due to the presence of a set of biologically active substances, especially in essential oil, which possesses a multidirectional therapeutic activity being used in the treatment of gastrointestinal, cardiovascular, respiratory, and urinary infections [6]. Scientific studies reported anti-inflammatory [7], antioxidant [8,9], sedative [10], cytotoxic [11,12], analgesic [7], antimicrobial [6,13,14], and anticonvulsive [15] properties of *L. angustifolia* essential oil. Literature data reveal a huge variation in terms of *L. angustifolia* essential oil content, with values ranging between 0.5 and 6.25% in the case of essential oil obtained from fresh and dry inflorescences [16]. The main constituents of *L. angustifolia* essential oil are linalool, linalyl acetate, 1,8-cineole, borneol, camphor, lavandulyl acetate, *β*-caryophyllene, *β*-ocimene, *α*-fenchone, terpinen-4-ol, caryophyllene oxide, limonene, pinenes, geranyl acetate, *β*-farnesene, santalene, lavandulol, camphene, geraniol, and *α*-terpineol [8,11,13,14,17,18,19,20,21,22,23,24,25,26]. The content of oxygenated monoterpenes prevails in *L. angustifolia* essential oil and varies between 36.33 and 92.90% [16].

The therapeutic effects of *L. angustifolia* are also determined by secondary metabolites such as oleanolic and ursolic acids, together with other pentacyclic triterpenes. [27,28]. It has been proven experimentally that both compounds in pure forms, as well as their synthetic derivatives, show multiple biological activities [29,30,31,32,33,34,35,36,37,38].

Some by-products, e.g., pomace or solid residues, that resulted after hydrodistillation of essential oil-producing plants could be considered as a source of biologically active compounds such as ursolic and oleanolic acids. In addition, residual distillation waters have various applications due to their aromatic and antimicrobial properties [39,40,41,42,43].

Antibiotic resistance is becoming one of the main problems of modern medicine since it substantially reduces the effectiveness of antibacterial treatments and is linked to increased patient mortality. As a result, known antibacterial preparations cease to be safe and effective against infections caused by resistant bacteria, leading to increasingly serious cases, including hospital-acquired complications. This requires the discovery of new classes of antibiotics or optimization and a combination of known compounds. However, microorganisms will likely evolve resistance in time and further research and development may be hard to sustain by the pharmaceutical companies. For this reason, studies are being conducted to identify effective remedies against multidrug-resistant strains. Preference is given to natural products among which are the essential oils [44], including lavender [45], or their combination with antibiotics [46]. Still, information about the antimicrobial activity of residual water and ethanolic extracts is very scanty and is mainly related to Lavander hydrosol, which is produced synthetically [47].

The aim of this study was to (i) evaluate the chemical composition of lavender essential oil and some of the waste by-products produced industrially in the Republic of Moldova using different chromatographic techniques; (ii) assess the in vitro antimicrobial activity of extracted compounds; and (iii) distinguish, using statistical analysis, between different lavender oils produced in different regions of the Republic of Moldova (Northern, Central, and Southern), based on the terpenic and aliphatic compounds.

## 2. Results

### 2.1. GC-MS Analysis Results

A total of 41 constituents of lavender essential oil were identified by means of gas chromatography-mass spectrometry (GC-MS) analysis (Table 1).

It must be mentioned that the essential oil with the richest content was made by producer P1, which is the largest and operates a stationary modern factory. By contrast, producers P2 to P7 use mobile installations and process raw plant material directly in the field, in modernized or artisanal installations, and this may influence the chemical composition of essential oils and resulting by-products.

According to the GC-MS data, the chemical composition of lavender essential oil produced in Moldova consisted mainly of terpenic and aliphatic compounds and their content varied within the limits indicated in Table 2.

The GC-MS analysis of extracts from residual waters (RW) showed that they contained only several hydrophilic components (see Section 3.3) and represented about 0.3–0.5% of the volume.

### 2.2. RP-HPLC Analysis Results

The content of triterpenic oleanolic acid (OA) and ursolic acid (UA) was established in freshly dried lavender plants and in dried solid residues (after hydrodistillation) via RP-HPLC analysis.

The results were expressed as mg/g for extracts and mg/100 g for the ratio plant material/solid residue (Table 3 and Table 4). It was observed that fresh plants had a much higher content of OA and UA.

The lower content of OA and UA in solid residues can be explained by their loss and derivatization/degradation during hydrodistillation in an aqueous medium at elevated temperatures (Table 4). The latter seemed more relevant since neither OA nor UA was found in residual water extracts (see Section 3.3).

### 2.3. Microbial Inhibition Assessment Results

The microbial activity assessment of lavender essential oil extracts from lavender plant material (LPM), lavender by-products (residual water (RW), and solid waste residue (SR)) was performed by serial dilution methods against several non-pathogenic Gram-positive and Gram-negative bacteria strains and fungi species, including phytopathogenic ones (e.g., *Xanthomonas campestris, Erwinia* *amylovora*, and *Erwinia carotovora*).

The results of the lavender essential oil antibacterial and antifungal activity tests are presented in Table 5.

The same method was applied for residual waters, ethanolic extracts from solid residues, and freshly dried lavender plant materials (Table 6).

All of the samples were preliminarily tested for their in vitro antimicrobial activity and antifungal effect against pure cultures of three species of fungi (*Aspergillus niger*, *Alternaria alternate, Penicillium chrysogenum*) and against Gram-positive (*Bacillus* sp.) and Gram-negative bacteria (*Pseudomonas aeruginosa*). Microorganisms were provided by the American Type Culture Collection (ATCC, USA). Caspofungin and Kanamycin were used as performance standards for testing the antifungal and antibacterial activities. The minimum inhibitory concentration values (MIC) for all the samples and standards are summarized in Table 6.

## 3. Discussion

### 3.1. Chemical Composition of Lavender Essential Oils

The essential oil manufactured by producer P1, destined for export, had the following physico-chemical properties: Density (20 °C)—0.8920 g/mL; refractive index (n^20^_D_)—1.4660, and optical rotation (*α*^20^_D_)— −7.0°.

The most multitudinous group of terpenic compounds are monoterpenes, which include C_10_-hidrocarbones (8.72–15.32%) and their oxygenated derivatives (69.0–83.83%). The main constituents of this group which determine the quality and genuineness of lavender essential oil, according to the International Standard [48], are (%): 1,8-cineol (eucalyptol) (<1.0), (*E*)-ocimene (4.0–10.0), (*Z*)-ocimene (1.5–6.0), linalool (25.0–38.0), camphor (<0.5), terpin-1-en-4-ol (2.0–6.0), *α*-terpineol (<1.0), linalyl acetate (25.0–45.0), and lavandulyl acetate (>2.0) (Table 1 and Table 2).

The content of sesquiterpene hydrocarbons and their oxygenated derivatives is reported to be within the limits of 3.09–12.83% and 0.19–1.26%, respectively. According to the same source [48], the most important sesquiterpenes are: *β*-caryophyllene (4.78%), (*E*)-*β*-farnesene (1.52%), and caryophyllene oxide (0.36%) (Table 1 and Table 2).

Aliphatic compounds are of lesser concentration (1.42–3.90%) and in [48] are mentioned: 1-octen-3-ol (0.33%) and octan-3-one (<2.0%) (Table 1 and Table 2).

### 3.2. Chemical Composition of Lavender Plant Material

For the selective extraction of ursolic and oleanolic triterpene acids from the lavender plant materials (LPM), the extraction yield varied between 8.83–9.94%, with the OA content between 13.43–19.09 mg/g and UA content between 33.28–60.82 mg/g. The content of OA and UA in dry (DW) LPM was in the range of 133.11–168.57 mg/100 g, and respectively 329.83–537.00 mg/100 g DW LPM (Table 3).

Moreover, the experimental results showed that the sum of isomeric OA and UA in LPM was about 5% of the DW, in a 1:3.7 ratio, confirming that lavender is a valuable source of natural OA and UA triterpene acids.

### 3.3. Chemical Composition of Lavender by-Products

The GC-MS analysis of etheric extracts of residual water (RW) proved that they contain hydrophilic monoterpenic compounds such as 1,8-cineol (eucalyptol, 6.31%), linalool oxide (3.08%), linalool (78.05%), terpin-1-en-4-ol (1.92%), and *α*-terpineol (10.64%).

HPLC quantification of UA and OA indicated that RWs did not contain OA and UA triterpene acids.

In the case of solid waste residues (SR), the average extraction yield was about 3.91%, with the OA content between 27.48–39.37 mg/g and UA content between 80.82–135.56 mg/g (Table 4). The isomeric OA and UA in DW SR ranged between 113.47–144.98 and 313.95–499.15 mg/100 g, respectively (Table 4), with their amount accounting to about 1% of DW, in a 1:3.1 ratio, indicating that lavender by-products are a promising source of OA and UA triterpene acids.

Our results are consistent with other literature data reporting DW of lavender SR values between 136.0–259.7 and 346.3–648.4 mg/100 g [49].

### 3.4. Antimicrobial Assessments

Phytopathogenic bacteria can cause various diseases of agricultural plants, especially the genera Erwinia and Xanthomonas. For example, *Erwinia amylovora*, the Gram-negative bacterium of the Enterobacteriaceae family, is the causative agent of fire blight, a devastating plant disease that affects a wide range of species of the family Rosaceae and is a major global threat to commercial apple and pear production. [50]. Another species, *E. carotovora*, causes bacterial soft rot in economically important crops, such as potatoes, tomatoes, and cucumbers. In the case of potatoes, the soft rot of the stem and tubers occurs even after harvest, thus considerably reducing the yield [51]. *Xanthomonas campestris* pv. *vesicatoria* is a biotrophic Gram-negative bacterium and is the agent that causes bacterial leaf scorch on tomatoes (*Solanum lycopersicum* L.) and peppers (*Capsicum annuum*), a disease that is present worldwide. Symptoms of bacterial infection include defoliation and chlorotic necrotic lesions on leaves, stems, fruits, and flowers, which subsequently lead to reduced fruit yield [52].

The species *Bacillus subtilis* and *Pseudomonas fluorescens* do not cause any disease to plants but were selected as reference bacteria from the Gram-positive and Gram-negative groups. They are also very suitable as test objects for evaluating the antibacterial activity of the lavender extract. *Candida utilis* and *Saccharomyces cerevisiae* are also non-pathogenic but were used as representatives of the yeast-fungus group for evaluating the antifungal activity of the extract.

It should be mentioned that there is a lack of information about any antimicrobial effects of lavender essential oil on *E. carotovora*, *E. amylovora*, and *C. utilis*.

The in vitro assessment of lavender essential oil of Moldovan origin showed good antibacterial activity against both non-pathogenic Gram-positive/Gram-negative bacteria (*B. subtilis* and *P. fluorescens*) at MBC of 300 μg/mL and good to high antifungal activity against phytopathogenic bacteria (*X. campestris*, *E. amylovora*, *E. carotovora*) and *C. utilis* fungi at MFC of 150–300 μg/mL (Table 5).

The highest antifungal and antibacterial activities were observed for residual water (RW) at 0.08 and 0.125 μg/mL, respectively. Good antifungal and antibacterial activities were ascertained for the SR extract as well (0.50 and 4 μg/mL). The LPM extract showed moderate antifungal and antibacterial activity (0.75 and 6 μg/mL).

The two techniques employed for testing both the disc diffusion and the dilution methods have been developed to yield accurate measurements of antibacterial and antifungal activities and are routinely used in antimicrobial susceptibility testing.

According to the obtained results, the antibacterial activity was similar but the antifungal activity was slightly different, thus suggesting that the activity against different microorganisms could be caused by different components of the oil.

### 3.5. Statistical Data Analysis

Univariate as well as multivariate statistical data analysis (SDA) represent one of the most reliable methods that permit extracting useful information and inferring different hypotheses concerning the considered set of data. Given the great diversity of organic compounds which can be found in lavender essential oil, multivariate statistical data analysis was an appropriate method allowing to group samples, in this case, according to the lavender oil producer and based on the concentrations of organic compounds (R mode), or, to classify an experimentally determined organic compound based on the concentration in samples (Q mode) [53,54].

**Figure 1:**
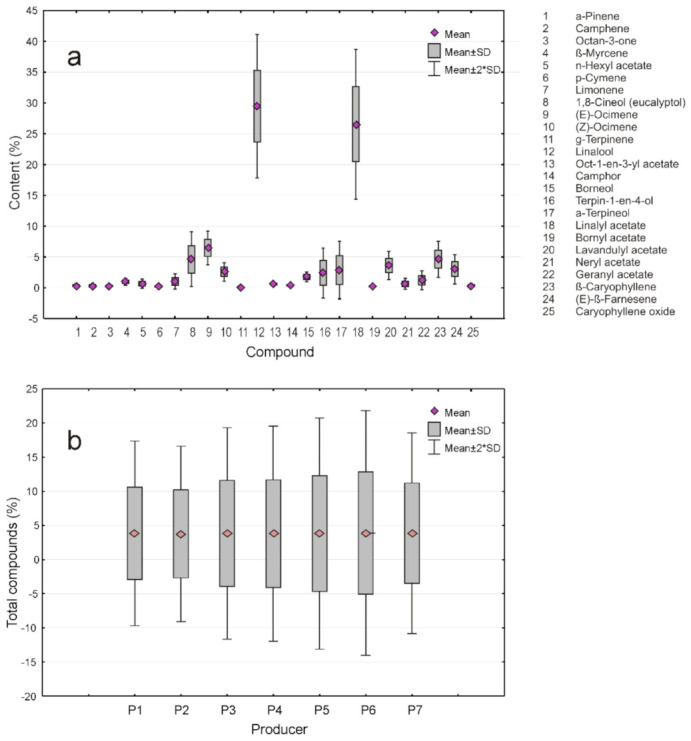
Box plots representing the distribution of (**a**) 25 components of lavender oil and (**b**) total content of compounds present in all the samples (producers).

It is worth mentioning that, to avoid any errors induced by missing data, SDA was applied only in the cases of compounds with a non-negligible variation present in all the samples (Table 1), i.e., the compounds which permitted generating the box plots in Figure 1a,b.

Univariate SDA was useful in establishing the extent to which the samples of lavender oil by the seven producers were similar. This information was obtained by analyzing the box plot shown in Figure 1a. It was observed that all the samples were quite similar. To confirm this, we used more univariate tests, such as one-way ANOVA, Tukey’s pairwise test, Kruskal-Wallis test of equal medians, as well as Mann-Whitney U tests. All of them confirmed that between the lavender oil samples there are no statistically significant differences. For this reason, we have proceeded with multivariate SDA.

Within multivariate SDA, each sample (case) is characterized by independent parameters (variables), so that the final analysis can be performed in R mode (to study relations between samples based on variables) or Q mode (to study the interrelations between variables based on samples). As both methods were based on the same set of samples and variables, R and Q modes could be considered complementary, which significantly enhanced the analysis.

Depending on the situation, cases/variables can be grouped by a multitude of procedures among which covariance and correlation are frequently utilized.

In the case of lavender samples, the best results were obtained by the principal component analysis (PCA) applied in both R and Q modes. With respect to the other two SDA methods, cluster analysis and K mean clustering, PCA permitted evidencing the association of samples, i.e., seven producers of lavender oil in R mode, as well as 25 lavender oil compounds in Q mode. Moreover, in R mode, a tree diagram corresponding to the cluster analysis (Euclidean distances) is, concerning the number and structure of clusters, similar to PCA based on correlation. For this reason, we restrained our SDA to both R and Q mode PCA.

**Figure 2:**
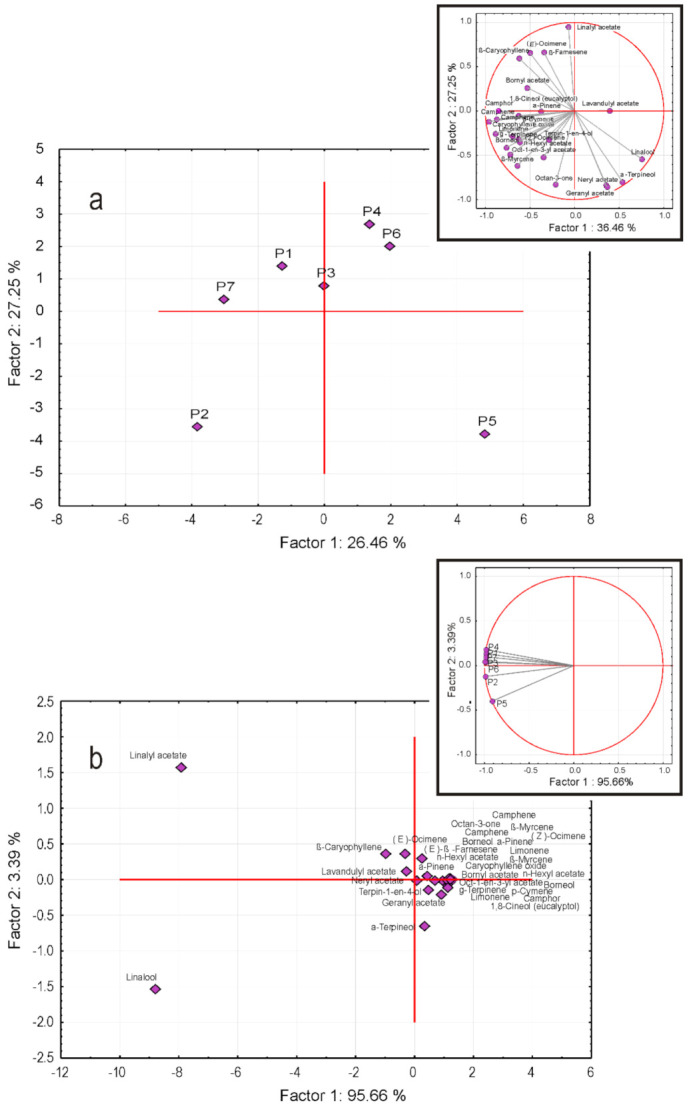
The results of R (**a**) and Q (**b**) mode PCA. The insets illustrate the contribution of the corresponding principal component (PC) analysis.

The results, represented by the principal component (PC) 2 vs. PC 1 bi-plots, are illustrated in Figure 2a,b, respectively. In both cases, the PCA was based on correlations between variables (organic compounds, R mode) or samples (lavender oil producer, Q mode). Moreover, the loadings of each variable or sample were represented by Factor 2 vs. Factor 1 bi-plots in the corresponding insets: Variables in Figure 2a and samples in Figure 2b.

**Figure 3:**
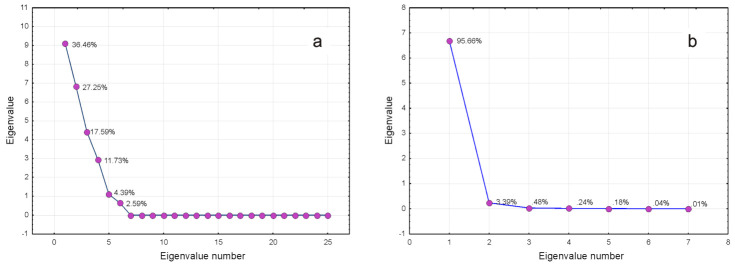
The screen plots corresponding to R-mode (**a**) and Q-mode (**b**) PCA.

Accordingly, the result of PCA in R mode is illustrated by the bi-plot in Figure 1a. The existence of at least three clusters can be remarked, two of which consist of only one member, i.e., producers P2 and P5, and a third one, grouping the rest of the producers. The bi-plot illustrating the contribution of each compound to the PC1 and PC2 showed a relatively balanced situation, as both Factors 1 and 2 had similar contributions to PC, consisting of 36.46 and 27.25%, respectively. It is worth mentioning that a similar result was obtained by considering the PC3 vs. PC2, which most probably could be explained by their contribution to the total variance, 25.25 and 17.57%, respectively. The corresponding screen plot in Figure 3a illustrated this finding.

Complementary to the R-mode, a Q mode PC2 vs. PC1 bi-plot, shown in Figure 2b, consisted of three clusters, two of which contained a single organic compound, i.e., linalyl acetate and linalool, while the third one included all other 23 compounds. This result was in good agreement with the composition of the investigated samples, according to which, both linalyl acetate and linalool were characterized by the highest concentrations and variances.

On the contrary, Factor 2 vs. Factor 1 (Figure 2b, inset), except for Producers 2 (P2) and 5 (P5), were nearly coincident and negatively oriented along the first axis, which suggested an almost equivalent contribution to the total variance. This finding may explain the fact that PC1 contributed about 96% to the total variance, as shown in the corresponding screen plot (Figure 3b). In this regard, it is of interest to remark, as mentioned before, that P2 and P5 formed two different uni-component clusters (Figure 2b).

## 4. Materials and Methods

### 4.1. Samples Collection

The samples of *L. angustifolia* vegetal raw material, by-products, as well as the main product—lavender essential oil (LEO), were provided between 2016 and 2018 by seven producers (P 1-7) from different regions of the Republic of Moldova (Northern, Central, and Southern): P1—Causeni district; P2—Donduseni district; P3 and P6—Rezina district; P4—Falesti district; P5—Dubasari district; and P7—Ungheni district.

For OA and UA characterization, fresh lavender inflorescences were collected directly from the lavender fields near the Pervomaisc village, Causeni district (46°42′04″ N 29°05′21″ E). The inflorescences were dried in shaded places to obtain lavender plant material samples (LPM) (n = 3) which were subjected to HPLC characterization. The by-products which resulted after hydrodistillation (solid residue—SR (n = 3) and residual water—RW (n = 1)), were collected from the factories, dried, and bottled.

### 4.2. Chemicals

All of the used solvents, reagents, and standards were of analytical grade. Anhydrous sodium carbonate, aluminium chloride, sodium acetate, 96% ethanol, methanol, diethyl ether, and petroleum ether were obtained from Merck (Darmstadt, Germany). Deionized water produced by a Milli-Q Millipore system (Bedford, MA, USA) was used for the preparation of aqueous solutions and UHPLC mobile phases.

The standards used for HPLC-PDA analysis (ursolic and oleanolic acids) were HPLC purity and purchased from Sigma-Aldrich (Steinheim, Germany). Stock solutions of all the standards were prepared in methanol. Working standards were made by diluting the stock solutions in the same solvent. Both stock and working standards were stored at 4 °C until further use.

### 4.3. Extracts Preparation

**Figure 4:**
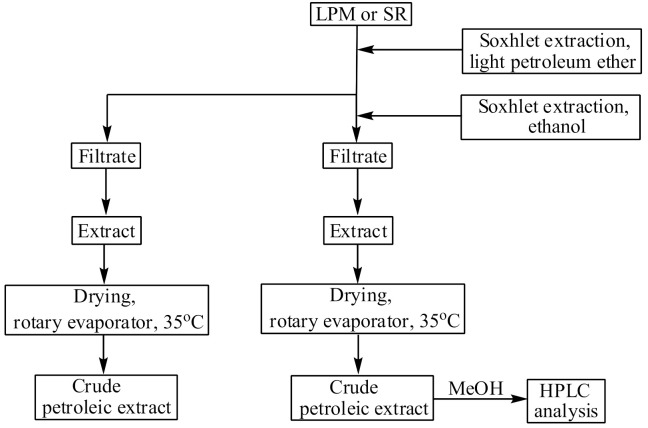
Flowchart of the UA and OA extract preparation.

The selective extraction of triterpenic ursolic and oleanolic acids with ethanol from the LPM and SR was performed using a Soxhlet type extractor after degreasing with light petroleum ether (b.p. 40 °C) (Figure 4.). The ethanolic extracts were evaporated to dryness at 35 °C under reduced pressure using a rotary evaporator. For HPLC analysis, aliquots of each crude extract were dissolved in methanol using ultrasonication and filtered through a 0.45 μm micro-filter. The extraction and HPLC analysis were performed in duplicate for each plant material and the results were expressed as a mean value. For GC-MS analysis, the industrially produced essential oil samples were dissolved in hexane. The RWs were extracted with diethyl ether and the obtained extracts were subjected to GC chromatographic analysis.

### 4.4. Analytical GC-MS Analysis

The GC-MS analysis was performed using an Agilent Technologies 7890A gas chromatograph coupled with a 5975C Mass-Selective Detector (MSD) equipped with a split/splitless injector (1 µL). The analysis was carried out on an HP-5MS fused silica capillary calibrated column (30 m × 0.25 mm i.d.; film thickness 0.25 µm). The injector and detector temperatures were kept at 250 °C. Helium was used as carrier gas at a flow rate of 1.1 mL/min; oven temperature program was 70 °C/2 min, which was then programmed to 200 °C at the rate of 5 °C/min, and finally to 300 °C at the rate of 20 °C/min. The split ratio was 1:50, the MSD ionization energy was 70 eV, scan time was 1 s, the acquisition mass was in the range from 30 to 450 amu, and the solvent delay was 3 min.

### 4.5. Analytical RP-HPLC Analysis

The ursolic and oleanolic acids were quantified by an HPLC-PDA method previously reported [55], using a Thermo Finnigan Surveyor Plus HPLC System (Thermo Fisher Scientific Inc., San Jose, CA, USA). The OA and UA from extracts were identified by their retention time and spectral data by comparison with standards. To confirm the peak identity among possible interference peaks, the technique of standard addition to the sample was applied. Moreover, the peak purity for the interest peaks was satisfactory.

Calibration curves of the standards covered the range of 1–400 mg/L for both OA and UA and revealed good linearity, with correlation coefficients higher than 0.995 (0.9989 for OA and 0.9991 for UA) [56]. The accuracy of the method (%) was evaluated for spiked samples at 50 mg/L concentration and the obtained average values were 4.31% for OA and 3.65% for UA.

### 4.6. Antimicrobial Activity Assessment

The in vitro antimicrobial activity tests of methanolic extracts from the SR and RW against three species of fungi (*Aspergillus niger*, *Alternaria alternata,* and *Penicillium chrysogenum,* ATCC 53346, 8741, and 20044) and two species of bacteria (*Pseudomonas aeroginosa* and *Bacillus* sp., ATCC 27813 and 15970) were performed using a previously reported method [57].

Antimicrobial activity assessment of the industrially obtained lavender essential oil samples was performed in vitro on the following microorganisms: Non-pathogenic Gram-positive and Gram-negative strains of *Bacillus subtilis* NCNM BB-01 (ATCC 33608) and *Pseudomonas fluorescens* NCNM-PFB-01 (ATCC 25323), phytopathogenic strains of *Xanthomonas campestris* NCNM BX-01 (ATCC 53196), *Erwinia amylovora* NCNM BE-01 (ATCC 29780), *E. carotovora* NCNM BE-03 (ATCC 15713), and fungus strains of *Candida utilis* NCNM Y-22 (ATCC 44638) and *Saccharomyces cerevisiae* NCNM Y-20 (ATCC 4117) following a method described elsewhere [58].

The compounds Caspofungin and Kanamycin, both from Liofilchem (Roseto degli Abruzzi, Italy), were used as standards for antifungal and antibacterial activity tests.

### 4.7. Statistical Analysis

All statistical data analyses were performed using the StatSoft Statistica 10 software.

## 5. Conclusions

More than 40 main constituents of lavender essential oil from seven Moldavian producers were quantified by means of chromatographic and statistical analyses. The experimental data for lavender plant material and solid waste residue proved the possibility of their use as sources of biologically active compounds, such as OA and UA triterpene acids. All of the subjects in the present study, essential oil, residual distillation waste water, and extracts from the solid waste residues have shown high antimicrobial activity against 11 strains of bacteria and fungi, including phytopathogenic ones.

## Figures and Tables

**Table 1:** Phytochemical (terpenic and aliphatic compounds) composition of lavender essential oil of Moldovan origin.

No.	RT* (min)	Component	Producer, Content (%)						
			P1	P2	P3	P4	P5	P6	P7
1	4.416	*α*-Pinene	0.36	0.57	0.36	0.18	0.09	0.26	0.57
2	4.710	Camphene	0.34	0.47	0.30	0.09	0.09	0.26	0.63
3	5.179	Sabinene	0.14	-	-	-	-	-	-
4	5.240	1-Octen-3-ol		0.83	-	-	-	0.15	0.27
5	5.263	*β*-Pinene	0.51	-	0.34	0.39	0.38	0.22	0.57
6	5.398	Octan-3-one	0.28	0.51	0.31	0.25	0.39	0.12	0.21
7	5.489	*β*-Myrcene	1.06	1.50	0.96	0.80	0.89	0.62	0.89
8	5.577	Octan-3-ol	0.13	0.18	0.20	0.17	0.20	-	-
9	5.962	*n*-Hexyl acetate	0.59	1.27	0.31	0.42	0.55	0.52	1.17
10	6.284	*p*-Cymene	0.22	0.46	0.22	0.14	0.10	0.24	0.39
11	6.400	Limonene	0.52	1.79	0.79	0.45	0.55	1.17	1.93
12	6.455	1,8-Cineol (eucalyptol)	5.00	3.81	2.22	3.73	4.44	3.83	9.29
13	6.574	(*E*)-Ocimene	8.06	5.87	6.85	7.86	4.37	5.25	7.15
14	6.807	(*Z*)-Ocimene	3.74	3.45	2.59	2.55	1.86	1.76	2.16
15	7.087	*γ*-Terpinene	0.10	0.29	0.29	0.06	0.07	0.07	0.15
16	7.443	Linalool oxide	-	0.17	0.07	0.11	0.12	-	-
17	7.824	*δ*-Terpinene	0.27	-	-	-	-	-	-
18	7.825	*α*-Terpinolene	-	0.58	0.25	0.21	0.32	0.16	0.23
19	8.238	Linalool	23.54	27.98	29.06	25.57	40.68	33.29	26.19
20	8.392	Oct-1-en-3-yl acetate	0.56	0.82	0.60	0.71	0.63	0.39	0.58
21	9.308	Camphor	0.47	0.47	0.37	0.36	0.30	0.32	0.61
22	9.847	Borneol	1.92	2.15	1.68	1.28	1.65	1.41	2.40
23	10.00	(3*E*,5*Z*)-Undeca-1,3,5-triene	0.17	-	-	-	-	-	-
24	10.15	Terpin-1-en-4-ol	1.30	4.65	5.98	0.94	1.67	1.41	1.03
25	10.41	Cryptone	0.29	0.29	-	-	-	0.30	0.33
26	10.50	*α*-Terpineol	2.42	3.31	2.02	1.42	7.95	1.49	1.61
27	11.49	Nerol	0.38	0.46	0.23	0.14	1.14	-	-
28	11.84	*p*-Cumic aldehyde	0.13	0.15	-	-	-	0.18	-
29	12.32	Linalyl acetate	26.55	20.26	28.65	32.25	16.68	33.30	28.10
30	13.01	Bornyl acetate	0.32	0.25	0.24	0.27	0.19	0.17	0.24
31	13.11	Lavandulyl acetate	4.88	2.84	2.36	4.83	4.78	2.56	3.07
32	14.98	Neryl acetate	0.78	0.91	0.39	0.33	1.53	0.31	0.37
33	15.47	Geranyl acetate	1.31	1.69	0.79	0.73	2.70	0.59	0.67
34	15.66	*α*-Zingiberene	0.15	-	-	-	-	-	-
35	16.47	*β*-Caryophyllene	6.25	5.33	4.62	5.44	1.64	4.93	4.32
36	16.80	*α*-Bergamotene	0.27	0.28	0.19	0.20	0.05	0.16	-
37	17.31	(*E*)-*β*-Farnesene	4.86	2.59	3.65	3.94	1.23	2.46	2.45
38	17.97	*β*-Cubebene	1.12	0.82	1.03	0.69	0.17	-	-
39	18.75	*γ*-Cadinene	0.18	0.53	-	-	-	0.68	0.65
40	20.39	Caryophyllene oxide	0.45	0.69	0.19	0.29	0.21	0.35	0.28
41	21.69	Cadinol	0.17	0.57	-	-	-	0.18	-
Total content, (%)			99.80	98.79	98.17	96.80	97.62	99.11	98.51

*RT: Retention time; P 1–7: Producers.

**Table 2:** Chemical composition of lavender essential oil.

Class	Subclass	Content, (%)
Terpenic compounds		94.89–97.77
	Monoterpenes	84.08–92.55
	Monoterpene hydrocarbons	8.72–15.32
	Oxygenated monoterpenes	69.00–83.83
	Sesquiterpenes	3.30–13.45
	Sesquiterpene hydrocarbons	3.09–12.83
	Oxygenated sesquiterpenes	0.19–1.26
Aliphatic compounds		1.42–3.90
	Hydrocarbons	0.17
	Alcohols	0.13–1.01
	Ketones	0.25–0.80
	Esters	0.91–2.09
	Total	96.80–99.79

**Table 3:** The OA and UA content of lavender plant material (DW).

Lavender Plant Material	Extract Yield (%)	Concentration (mg/g Extract)		Concentration (mg/100 g Lavender Plant Material, DW)	
		OA	UA	OA	UA
LPM 1	9.94	16.19	37.46	160.95	372.36
LPM 2	8.83	19.09	60.82	168.57	537.00
LPM 3	9.91	13.43	33.28	133.11	329.83

**Table 4:** The OA and UA content of lavender by-product (solid waste residue), (DW).

Lavender by-Product (Solid Residue, SR)	Extract Yield (%)	Concentration (mg/g Extract)		Concentration (mg/100 g Dry Solid Residue)	
		OA	UA	OA	UA
SR 1	3.88	29.21	80.82	113.47	313.95
SR 2	3.68	39.37	135.56	144.98	499.15
SR 3	4.15	27.48	87.90	114.07	364.89

**Table 5:** The antimicrobial activity of lavender essential oil.

MBC and MFC, μg/mL						
Sample	*Bacillus* *subtilis*	*Pseudomonas* *fluorescens*	*Xantdomonas* *campestris*	*Erwinia* *amylovora*	*Erwinia* *carotovora*	*Candida* *utilis*
LEO	300	300	300	150	300	150

MBC: Minimal bactericidal concentration; MFC: Minimal fungicidal concentration.

**Table 6:** The antimicrobial activity of residual water and ethanolic extracts from lavender plants.

Sample	MIC (μg/mL)				
	*Aspergillus niger*	*Alternaria alternata*	*Penicillium chrysogenum*	*Bacillus* sp.	*Pseudomonas* *aeruginosa*
Residual Water	0.08	0.08	0.08	0.125	0.125
Extract from SR	0.50	0.50	0.50	4	4
Extract from LPM	0.75	0.75	0.75	6	6
Caspofungin ^a^	0.24	0.24	0.24	-	-
Kanamycin ^b^	-	-	-	3.5	3.5

RSD (μg/mL): ^a^ ±0.001 ^b^ ±0.0002.

## Abbreviations

ATCC	American type culture collection
DW	Dry weight
DW PLM	Dry weight lavender plant material
GC-MS	Gas chromatography-mass spectrometry
HPLC	High-performance liquid chromatography
HPLC-PDA	High-performance chromatography-photodiode array detection
LEO	Lavender essential oil
LPM	Lavender plant material
MBC	Minimum bactericidal concentration
MFC	Minimum fungicidal concentration
MIC	Minimum inhibitory concentration
NCNM	National Collection of non-pathogenic microorganisms
OA	Oleanolic acid
RP-HPLC	Reversed-phase high-performance chromatography
RSD	Relative standard deviation
RW	Residual water
SDA	Statistical data analysis
SR	Solid residue
UA	Ursolic acid
